# Entrepreneurial metacognition: a study on nascent entrepreneurs

**DOI:** 10.1007/s11365-022-00799-1

**Published:** 2022-08-15

**Authors:** Bob Bastian, Antonella Zucchella

**Affiliations:** Department of Economics and Management, Universita of Pavia, Via Felice Cavallotti 6, Pavia, 27100 Italy

**Keywords:** Entrepreneurial cognition, Metacognition, Decision-making, Social capital, Teams

## Abstract

This paper contributes to uncovering the role of metacognition in the decision-making process of entrepreneurs. Specifically, we analyze nascent entrepreneurs in their process of start-up development while relying on metacognitive processes. The experiences of a sample of new venture initiatives are explored in two distinct phases, a start-up competition and the subsequent launch of their venture. Following the Gioia protocol, the study contextualizes the process in which social capital reinforces metacognitive processes. This process stimulates nascent entrepreneurs to consider alternatives, such as extending expertise outside the start-up. Moreover, we find that these processes support entrepreneurs and their teams in improving their decision-making processes. The findings support that nascent entrepreneurs rely heavily on the input of others in their start-up creation process, and contribute to new empirical insights about entrepreneurial metacognition. A dynamic model in which these relationships emerge is developed. The study’s results contribute to a better understanding of the antecedents and consequences of metacognitive processes in nascent entrepreneurship.

## Introduction

In their decision-making process, entrepreneurs depend on incomplete, sub-optimal circumstances with varying levels of uncertainty, ambiguity, time pressure, and emotional stress (Forbes, 2005; Packard et al., 2017). To piece together previously unconnected information, entrepreneurs use their mental models to keep moving between intuitive and reflective cognitive processes (Mitchell et al., 2003; Zollo et al., 2017). These models, even when incomplete, help entrepreneurs identify opportunities and start ventures under conditions of uncertainty. Fast and frugal intuitions, however, can lead to cognitive traps in decision-making (Abatecola et al., 2018; Busenitz & Barney, 1997; Simon, 2000). Given that it is in an entrepreneurs’ best interest to make deliberate decisions, it is fundamental to develop accurate self-perceptions about one’s decisions through a rational system of thought (Sadler-Smith, 2016). Following this line of reasoning, entrepreneurs may benefit from metacognitive processes, because they increase self-awareness, promote accurate judgment, and help to identify when one might be erroneous in their decision-making (Kruger & Dunning, 1999).

The process of an individual’s understanding and knowledge of their own cognition, is known as metacognition (Flavell, 1979). Metacognition refers to ‘thinking about thinking’ and represents one’s conscious reflection about one’s thinking (Schraw & Dennison, 1994). To think ‘metacognitively’ describes activities such as “to be self-aware, to think aloud, to reflect, to be strategic, to plan, to have a plan in mind, to know what to know, to self-monitor” (Guterman, 2002, p. 285). Whereas cognition is required to complete a task, metacognition is needed to understand how a task will be performed (Akturk & Sahin, 2011; Schraw, 2001). Individuals that effectively apply metacognition have been shown to create self-benefits such as accurately estimating and updating their knowledge, and monitoring and evaluating on-going learning, because metacognition triggers analytic reasoning to evaluate and refine intuitive reasoning (Alter et al., 2007; Everson & Tobias, 1998).

Metacognition has received attention by entrepreneurship scholars only recently. For example, Haynie et al. (2010) suggested foundations of the entrepreneurial mindset to be metacognitive by nature because metacognitive processes are contextual responses to novel and dynamic contexts. However, our current understanding of metacognition is incomplete. Specifically, more research is needed in exploring “metacognitive aspects on entrepreneurial decision-making” (Shepherd et al., 2014, p. 24). Contextual factors that influence entrepreneurs and their metacognitive processes are scarce and need further exploration in the entrepreneurial decision-making process (Haynie et al., 2012). For example, metacognitive processes do not only require awareness of the self, but also of others’ thinking with the help of observations and interactions (King, 1998). Moreover, when individuals reason, metacognitive processes promote ‘a second opinion’ that activates more analytic, slow, and logical reasoning in the decision-making processes (Graber et al., 2012). These preceding factors are important to understand because entrepreneurs have been shown to rely heavily on their social environment, and ability to make well-informed decisions in their start-up process (Shepherd et al., 2014). However, little entrepreneurial research has addressed these research gaps. Specifically, explorative research in metacognitive studies has mostly been neglected so far.

To address these gaps, this paper explores how entrepreneurs use the understanding of their cognition to develop their start-ups. In addressing this question, this study adopts an explorative lens and analyzes how a sample of nascent entrepreneurs cognitively handles their decision-making process while coping with different contexts of uncertainty. Moreover, although previous studies have given fruitful results using quantitative methods, this study explores contextual factors that emerge during the start-up process, providing methodological novelty by applying a qualitative approach to better understand metacognition as a “multifaceted phenomenon” (Efklides, 2008, p. 277; Mitchell et al., 2014). Following the Gioia protocol, this study emphasizes the practical utility of the heterogeneous ways entrepreneurs employ their metacognition when they undertake start-up activities (Corley & Gioia, 2011).

The results of this study highlight a central role for entrepreneurs’ metacognitive processes while capitalizing on their social capital. Specifically, the study provides an alternative perspective on the role of social capital for nascent entrepreneurs, emphasizing that metacognitive processes are stimulated by others. Metacognitive processes lead nascent entrepreneurs to go beyond their social networks, structures, and memberships to search for expertise and engage with outsiders. These interactions stimulate entrepreneurs to cognitively adopt feedback, either from the extent to which they leverage human relationships inside and outside their venture, or from the extent in which they benefit from comparison within their social structures. Additionally, we find these metacognitive processes to advance entrepreneurs and their teams in improving their decision-making processes. Metacognitive processes appear to have a significant effect particularly on team composition, because differentiated teams increasingly stimulate these metacognitive processes during the start-up creation process. The findings highlight the need for entrepreneurs new to the start-up process to develop metacognitive processes because it supports the mobilization of resources in order to engage in further thinking about thinking processes to achieve business growth. At the same time, we report dropout cases from the start-up process when metacognitive processes are not enough developed.

This study contributes to theory, providing a processual model in which is shown how and which social capital elements play a central role in the development of metacognitive processes for individuals and teams new to the entrepreneurial start-up process. The model further explains how metacognitive processes stimulate nascent entrepreneurs to move beyond their status quo by expanding their local ecosystems and utilizing expertise in order to add cognitive resources to the start-up. These advantages support entrepreneurs and their decision-making processes.

Additionally, the findings of this study highlight relevance for practitioners and policymakers in educational settings and incubator programs, since metacognition can be strengthened by experience and training (Nelson, 1996; Nietfeld & Schraw, 2002).

The remainder of the present study is structured as follows. The next section briefly draws a theoretical framework of metacognition, followed by the research method and the empirical section. The final section of the study suggests theoretical and practical implications, limitations, and interesting new directions for future metacognition research in entrepreneurship.

## Theoretical framework

Metacognition in start-up creation processes is a crucial cognitive enabler to predictor entrepreneurial intentions and leads to superior performance (Botha & Bignotti, 2017; Dew et al., 2015; Urban, 2012). In their decision-making process, metacognition is particularly important to be developed in the early stages for entrepreneurs because it helps to monitor and cope with uncertainty (Qiu et al., 2018). Metacognition, which refers to an individuals’ understanding and knowledge of their own cognition, has been defined as the ability to reflect upon, understand, and control cognitive processes relating to a concrete goal or objective (Flavell, 1976; Schraw & Dennison, 1994). These benefits help entrepreneurs who make better use of their metacognition to use cognitive feedback more effectively (Haynie et al., 2012). On the contrary, those that are restricted in their metacognitive abilities are less likely to show cognitive flexibility within a changing environment (Earley & Ang, 2003).

Metacognitive processes facilitate entrepreneurial expertise because of its self-regulating nature. This is because metacognition consists of knowledge about cognition and self-regulatory mechanisms that help entrepreneurs in their learning process to plan, monitor, and reflect (Fust et al., 2018; Mitchell et al., 2007). “To successfully self-regulate, people need to be aware of their goals and monitor and control their cognition”, and metacognition is “instrumental in this process” (Efklides, 2008, p. 282). Consequently, these processes stimulate individuals to obtain entrepreneurial expertise more quickly (Mitchell et al., 2006). For example, when entrepreneurs gain expertise, they are able to use their metacognition in a variety of ways and translate this into different types of opportunities. As a result, this helps entrepreneurs to identify and adapt to the cognitive nature of opportunities (Gustafsson, 2004).

Metacognition also helps individuals recognize certain elements about tasks and situations that enable effective and adaptable cognitive functioning when confronted with input from dynamic, or complex, environments (Brown & Eisenhardt, 1997; Flavell, 1979), actions typical of the venture creation process (McMullen & Dimov, 2013). This is important to understand because entrepreneurial success is influenced by the willingness to show adaptability towards a changing environment (Ireland et al., 2003; Shepherd et al., 2007). For example, metacognition helps entrepreneurs self-generate different frameworks, and combine them with a set of goals to make use of a changing environment (Haynie & Shepherd, 2009). Similarly, higher degrees of metacognition contribute to higher degrees of responsiveness to uncertainty (Mattingly et al., 2016). This explains why some entrepreneurs change their cognitive response to act and mobilize to a changing environment while others do not (Haynie et al., 2010).

More recently, new perspectives in metacognitive research reconsider the original facets of metacognition (Flavell, 1976) and highlight the relationship between metacognition and novel topics such as affect, social interaction, and decision improvement (Croskerry et al., 2013; Efklides, 2008; Koriat, 2007). For example, metacognition exists within social interactions that require awareness of not only the self but also of others’ thinking (Efklides, 2008; King, 1998). This is because metacognition can be seen as a multifaceted process that is continuously updated with the help of one’s awareness, monitoring of cognition, observation of the behavior of others, and interaction with different individuals (Efklides, 2008). These social dimensions of metacognition are particularly interesting for entrepreneurs in their start-up process, because aside from moments of isolated, independent thinking, the majority of their work involves direct, or indirect, interactions with other people (Foss & Grandori, 2020).

Moreover, metacognition operates as an analytic tool for cognitive de-biasing in order to improve decision-making processes. For example, metacognition may “serve as an alarm that activates analytic forms of reasoning that asses and sometimes correct the output of more intuitive thinking” (Alter et al., 2007, p. 569). This correction may be of particular interest for entrepreneurs and their susceptibility to cognitive biases resulting from intuitive reasoning, such as availability, a tendency to use information that comes to the mind most quickly, and confirmation bias, a tendency to favor information that confirms existing hypotheses (Barbosa & Fayolle, 2010; Bergen & Bressler, 2018). For example, metacognitive training helps individuals to overcome availability and confirmation bias because it stimulates a cognitive process in which to analytically perceive every decision scenario from multiple perspectives (Chew et al., 2016). Based on dual-process literature (Stanovich & West, 2000) in which individuals reason between two systems, either intuitive and automatic (labelled as system 1), or slow and analytic (labelled as system 2), metacognition is likely to play a role in the activation of slower, effortful, analytical processes (system 2) and as a result improve cognitive performance by reducing the impact of heuristics and defaults in judgment (Croskerry, 2000; Schwarz, 2015). This is because decisions are often made intuitively and unconsciously, and metacognition counteracts “the pernicious tendencies that drive people’s behavior” (Colombo et al., 2010, p. 446). For example, the extent to which managers make erratic strategic decisions is partially decreased by metacognition, because it enables individuals to reflect consistently in their decision-making process (Mitchell et al., 2011).

The preceding discussion emphasizes that metacognition is a multifaceted concept in which social interactions with different individuals need to be considered during entrepreneurial decision-making processes. Additionally, these perspectives underline an exploration of emerging concepts in the contextualization of metacognition. As a result, the following RQ has been developed for this study: How do entrepreneurs use the understanding of their cognition to develop their start-ups?

## Method and procedures

We adopt a theoretically grounded research approach as the method of this study, namely, grounded theory (Glaser & Strauss, 1967). To explore how entrepreneurs use the understanding of their cognition to develop their start-ups, the Gioia protocol has been chosen (Gioia et al., 2013). The Gioia protocol allows one to analyze social and psychological processes, through understanding the essence of individual experiences, and the processes by which it unfolds through emerging concepts (Gioia et al., 2013; Langley, 1999). The steps of the Gioia protocol start with the 1^st^-order analysis in which categories emerge from the interviews. Then, similarities and differences among these categories progress that is then labeled. Next, 2^nd^-order themes emerge from concepts and tentative relationships in which particular attention is focused on novelties and concepts that ‘leap out’. Finally, these themes are then further developed into 2^nd^-order aggregate dimensions that form together the basis of a data structure (Gioia et al., 2013, p. 20).

The Gioia protocol with semi-structured, in-depth interviews is suitable for the context of this study because it allows the researcher to (1) take advantage of emergent unpredictable issues, and (2) create an opportunity to dynamically respond, and elaborate upon, participants’ answers while generating new conceptual insights (Anderson et al., 2009; Cannatelli et al., 2019). Specifically, grounded theory is an appropriate method to use for this study since it supports uncovering relatively unexplored phenomena by means of emerging constructs, propositions, and process models (Glaser & Strauss, 1967). Although most studies of metacognition employ quantitative methods (e.g. Haynie et al., 2012; Mitchell et al., 2006, 2011), qualitative approaches, such as the use of semi-structured interviews, fit this study because “much can be understood about cognition and its metacognitive regulation through qualitative analysis” (Pressley, 2000, p. 261).

In this study, patterns have been found that represent interrelationships with, and between, metacognition. Additionally, this study analyzed an emerging process by assuming that behavior proceeds and constantly changes from complex interactions between the environment and the mind (Bruner, 1990; Fiske & Taylor, 2017). This has been untangled by moving between a continuous comparison of categories that arise from the interviews, and with the help of memo writing (Birks et al., 2008; Gioia et al., 2013; Glaser & Strauss, 1967). The transformative process of constant comparison has been crucial to study metacognition because “cognitive research places a noted emphasis on how, when, and why interactions between mind and environment play a role in the development, transformation, and use of mental representations and other cognitive constructs, and on how, when, and why these elements come to influence (and be influenced by) human action” (Grégoire et al., 2011, p. 1146–1147). The data structure that follows from this process, as portrayed by Corley and Gioia (2011), can be found in the finding section, while an empirical model is discussed in the discussion section, following Gioia et al., (2013).

## Sampling procedure and empirical context

This research adopts a longitudinal approach to study nascent entrepreneurs, throughout the process of launching a business. The context of this study can be divided into two distinct phases. The first phase refers to a start-up competition (SUC), in which nascent entrepreneurs present their intentions to start a business. The second phase represents the period after the SUC, in which entrepreneurs launched and expanded their businesses. The nascent entrepreneurs that were approached survived two preliminary rounds of a start-up competition with three jury experts as external evaluators, and were selected as the most promising candidates to participate and succeed in the competition. This study argues the choice for purposeful sampling as a suitable technique because the context belongs to a process of discovery on the individual level in entrepreneurship “information rich” studies (Birkner, 2020; Patton, 1990, p. 169). In total, 99 individuals applied to the SUC. Fifteen entrepreneurs were contacted and eleven of them agreed to be interviewed to become part of phase one of the study.

Start-up competitions are becoming an increasingly common context to investigate entrepreneurship because they produce new venture ideas, they have become increasingly well organized, and generate the birth of new firms (Schwartz et al., 2012; Watson et al., 2018). SUCs also facilitate entrepreneurial learning settings close to “very real business situations”, because participants develop a range of individual qualities and attributes that involve reflecting, an important goal of this study (Taylor & Thorpe, 2004; Wen & Chen, 2007, p. 361). The SUC used for this study has been structured by an organization consisting of experts and business angels, which has produced several classes of entrepreneurs over the years (some of which have launched successful businesses).

The goal of the competition for this study was to form a team, write a business plan, engage with investors, and eventually compete for an investment and a position in an incubator. In this phase, the nascent entrepreneurs of this study have intended to found their start-up, and this is where preliminary observations took place during several recruitment and pitch events (see Table 1). At the end of the SUC, a final pitch round took place where some of the interviewed participants received funding. Details about the entrepreneurs and their start-ups can be found in the Appendix (Table 2) where they have been grouped from C1 to C11. Since the purpose of a SUC serves to understand start-up intentions, as a next step, this study was particularly interested in determining if these entrepreneurs effectively developed their start-up after the SUC. In this second stage of our study, seven nascent entrepreneurs that were previously interviewed agreed to be interviewed again, while four dropouts were noted. Taking together stage one and stage two, 18 interviews were conducted for this study.

**Table 1:** Details on data collection

Source of data	Type of data	Use in the analysis
Observations	Start-up competition fair An all-day ‘recruitment’ event that took place on 21 October 2019 at a Technology Center. Each start-up that was invited for the competition presented the product/service at a desk with promotion materials In the following days, the start-ups formed a team to write a business plan Smart Building Expo A start-up event that took place on 15 November 2019. One of the most promising start-ups was here to find new potential customers Start-up competition final The final presentations with the most promising findings have been presented by each start-up during an event that took place on 16 December 2019. An investment of €20,000 has been awarded to the winner	We observed entrepreneurs, compared the process, and focused specifically on the preparation, presentation, and the way how the entrepreneurs interacted. Triangulated the data with field notes.
Interviews	First round Took place between September and December 2019. 11 interviews have been conducted with the founder(s) of start-ups [C1] – [C11]. Interviews were accompanied by field notes and had an average duration of 53 min. The data has been recorded and transcribed resulting in a total of 63 pages. Second round Took place between March and May 2020. 7 interviews have been conducted with the founder(s) of start-ups [C1], [C2], [C3], [C5], [C9], [C10], and [C11]. Interviews were accompanied by field notes and had an average duration of 76 min. The data has been recorded and transcribed resulting in a total of 64 pages.	We collected data regarding the interpretation of decision making processes, motivation, experience, team perception, uncertainty, and re-thinking processes. We compared the COVID-19 context as circumstances under high uncertainty, refined our theoretical assumptions and contextualized cognitive patterns.
Archival data	Application data of the start-up competition Data was collected in September 2019 at the initiation of the start-up event. A total of 99 start-ups expressed their interest to take part in the program. (1)We collected names of the entrepreneurs and their start-ups, age, industry, nationality, emails, LinkedIn profiles, areas of expertise, key competence, patent, and website. (2)Additionally, applicants had to fill in open questions such as ‘describe your idea’, ‘what is your main result so far’, and ‘why is your idea relevant’. Updates via emails Regular email traffic has been maintained between the researchers and the entrepreneurs. Pitch videos 10 videos (1 entrepreneur could not make it) have been produced where the interviewed start-uppers briefly introduced their start-up. Presentations Pitch and final video presentations were analyzed.	We triangulated observations and facts. Particularly, we paid attention to the process of each entrepreneur while participating in a semester start-up event, compared this process with the second round of interviews where entrepreneurs did not have specific incentives from a start-up competition investment.

Sample size in qualitative works has been debated widely without a clear consensus (Guest et al., 2006). This study follows Kuzel (1992, p. 41) who recommends working with a sample size between twelve to twenty “when looking for disconfirming evidence or trying to achieve maximum variation”. For example, Rashid and Ratten (2020) recently conducted 12 interviews using the Gioia protocol to contribute to new insights about an entrepreneurial phenomenon. During both interview phases, the order, and format of questions were flexible to maximize variation between respondents. The analysis and discussion sections of this study additionally focus on explaining the effects of individual differences on experience, on the explanation of dropout cases for those who abandoned business (and were not reachable for a second interview) during the process, and on contextual differences between the first and second phase. A structure with the main interview questions can be found in the appendix (Table 3).

## Participants

All entrepreneurs in this sample were considered to be nascent, because they all confirmed during interviews that (1) the concept of moving their idea toward a profitable business was relatively new to them and, (2) the potential investment was a crucial condition for moving forward after the SUC (Davidsson, 2006; Johnson et al., 2008). Nascent entrepreneurs are known to devote a significant amount of time and resources to the process of founding a firm. While latent, potential, and intentional entrepreneurs are mostly thinking about starting a business, nascent entrepreneurs and mainly concerned with the valorization of the opportunity by preparing a business plan and searching for investments, and therefore more advanced in the process (Passaro et al., 2017). The interviewed entrepreneurs of this study represent a key idea behind nascent entrepreneurship research, that is, the process of generating a sample of ongoing start-up efforts. The entrepreneurs were located in a dynamic entrepreneurial region with one of the highest GDP per capita ratios in Europe (Eurostat, 2020). In addition, the respondents varied according to age (21–41), sector (e.g. sustainable energy, food & beverages, biotech), and previous experience.

## Data collection

The data was primarily drawn from two main sources, namely (1) observations, and (2) semi-structured interviews. Additionally, to have a more comprehensive understanding of the data, this study integrated different sources such as application data, pitch videos, presentations, email updates, and field notes that supported the process of data collection. These sources are also supported by archival data. Following Rindova et al. (2011), Table 1 presents details about the source, type, and use of the data collection process. During the interviews, the questions of this study emphasized the relationship with metacognition as the literature suggests, which was when subjects responded with statements such as: “I was thinking”, “I was noticing”, “I was wondering”, “I was feeling”, “I knew what I had to know”, “I thought about that again”, or “Reflecting on that” (Guterman, 2002, p. 285). To obtain deeper insights, these answers were often followed up with questions such as: ‘can you give me an example to make me understand that better?’ or ‘could you tell me a bit more about that?’ In order not to constrain participants, they were not exposed to preconceived definitions or explanations of metacognition. In particular, the second step in the interviewing process allowed for a comparison between the entrepreneurs to observe the emergence of metacognition under different states of uncertainty. Every interview has been followed up with the production of a transcript within the timeframe that literature suggests (Corley & Gioia, 2011; Gioia et al., 2013).

## Findings

**Fig. 1:**
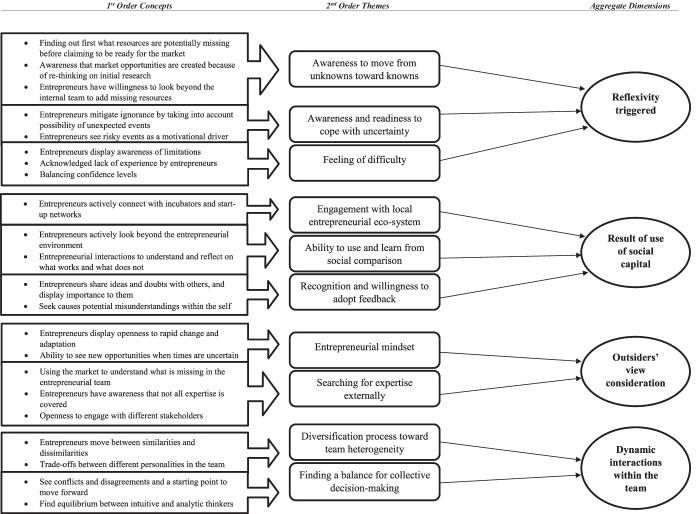
Data Structure

In this section, the study reports the outcomes of the research question: How do entrepreneurs use the understanding of their cognition to develop their start-ups? Following the Gioia protocol, informants’ quotes have been coded and collapsed into first order concepts, providing a first representation of their interviews outcomes. From first order concepts, second order themes have been developed, followed by aggregate dimensions, which represent the highest level of abstraction. Overall the three level of outcomes are portrayed in what Corley and Gioia (2011) denominate the ‘data structure’, represented in Fig. 1*.*

The four aggregate dimensions emerging from the empirical study refer to the role of the following issues in metacognitive processes of nascent entrepreneurs: triggers of the reflexivity of actors, use of social capital, consideration of outsiders’ view and interactions within the entrepreneurial team.

### Reflexivity triggered

The findings in Fig. 1 show that reflexivity of actors is triggered by (a) an awareness to move toward the ‘knowns’, (b) an awareness and readiness to cope with uncertainty, and (c) feelings of difficulty. These are individual processes of reflexivity that emerge from metacognition. Specifically, metacognition is conflated with reflexivity, in which aware individuals “test alternative solutions and reflect on differing outcomes” (Haynie et al., 2010, p. 220). Nascent entrepreneurs operate in an environment where a lack of expertise leads them to be ignorant of future outcomes, the so-called unknowns (Niittymies & Pajunen, 2019). These unknowns, such as selecting the right customer segmentation strategy, can become knowable with moments of reflection over time (Grant, 2021). By reflecting and analyzing upon their thinking, it may be possible for nascent entrepreneurs to discover what is already known to them, and what is not yet known. For example in the case of [C5]^1^: *I think, everybody has its own conception. Some people are more cautious, other people move more on intuition, and they are immediately enthusiastic. By nature we, scientists, tend to be cautious for we make the next step. I should be really, really confident and convinced that my product is the best.*

An intuitive thinking style often leads to satisfying outcomes, but may occasionally lead to thoughtless choices (Kahneman, 2011). When more time is needed to make a decision, such as in the product development process of [C5], thinking about thinking processes may help entrepreneurs to become aware of unknowns and analyze the problem properly: *So this is all very new, we are all moving on a ground that is unknown for us. We have to understand things properly before proposing. In some cases, by intuition, we thought that a component would work. However, that was not exactly how it went, we realized it could be done in another way. When we realized that, we all agreed, and then went back to reprogram. ‘Now, let’s do things in another way’.*

That is because metacognitive processes, such as the feeling of difficulty, emerge when entrepreneurs move from intuition toward an analytic approach (Efklides, 2008). In this case [C5], it is recognized by an analytic approach that is preferred over an intuitive approach. The outcome of this process is the decision to reprogram the technology because moments of difficulty were experienced that made [C5] take a step back with the team. Moments of reflection help entrepreneurs anticipate future outcomes because it stimulates become aware of a set of alternative possible uncertain outcomes (Graber et al., 2012). This is the case when [C9] states that: *From a general point of view, I mean, it’s* (COVID-19) *a big mess. But at the same time, there are several opportunities that I think can somehow help this moment. So we are now thinking about how to adapt our sparkling sake to that concept. So, let’s say it is an ‘excuse’ to innovate. Me and a group of business partners, exploit the situation to launch a new product. So, in a couple of weeks, we are going to be out on Kickstarter, with this really innovative and new thing. I am really lucky that I have a group of people around me, we continuously think about our ideas.*

Extreme peaks of uncertainty, such as the COVID-19 pandemic, have been challenging for entrepreneurs. The results show that perceived feelings of difficulty were followed by reflective processes for those who were used to this practice. This habit of reflexivity leads to the discovery and creation of new business opportunities. Additionally, feelings of difficulty are important to embrace, says [C1]: *Maybe, if you want something so hard, you cannot see the difficulty.* Having, at the minimum, an awareness of these difficulties indicates self-reflection (Alvesson & Spicer, 2012). Those who practice self-reflection appear to be more prepared to cope with uncertain moments and difficulties. This is confirmed by the following case [C10]: *Before [N] joined the team, I kind of had a black hole answering business-related questions, in my mind, I could see the structure far away, but I could not focus on that.* (With [N] on board) *we told each other: ‘if we don’t act now, we will lose the idea’.* [C10] shows that reflection appears to be a powerful tool in moments where entrepreneurs may experience too much difficulty. It reveals that uncertainty about the business may be compensated by the extent to which entrepreneurs utilize thinking about thinking processes. However, coping with uncertainty is not axiomatic for nascent entrepreneurs, causing cognitive dissonance in the start-up creation process. This is confirmed by dropout case [C7]: *Something I don’t like, is that every day something is changing. So before we said, ‘let’s work on it in this way.’ The day after, we are changing it in a completely different way. Sometimes it is good because you can find a lot of new opportunities, but other times it is really annoying. You are working but you need to hold on all the time.*

### The use of social capital

Social capital emerges as an aggregate dimension capable of stimulating the metacognitive processes of entrepreneurs. Anderson and colleagues discuss the different conceptualization of social capital and conclude that social capital is “a social relational artefact, produced in interactions but that it resides within a network” (Anderson et al., 2007, p. 249). The metacognitive processes emerging from the use of social capital have the capacity to (a) stimulate the ability to learn from others, (b) stimulate the recognition of feedback, and (c) engage entrepreneurs with local ecosystems. Social capital, the ability of individuals to exploit benefits from their social networks, structures, memberships, emerges as a crucial antecedent of metacognitive processes for nascent entrepreneurs. In the context of this study, entrepreneurs have opportunities to pitch ideas during several events, while others are part of a scientific team or an incubator. These networks and structures emerge as an antecedent to stimulate metacognitive processes. For example, [C11] states that this was valuable to him in the development stage of the start-up because he did not consider before that feedback generates re-thinking processes in favor of the development of his start-up: *What I need is someone that is able to put you in the right way, someone that gives you feedback during the project. I didn’t think about that before.*

Another entrepreneur [C1] states that interactions help select the most valuable feedback. *The main challenge is the trade-off to listen to the feedback and consider their ideas without being narrow-minded. It is then up to us to make a good balance between that.* Social capital emerges as a reflective mechanism for entrepreneurs to think, re-think, select, and consequently adopt received feedback to improve start-up activities. Specifically, feedback emerges as a major driver of motivation and business improvement, because it is the input of others that stimulates new thinking patterns.

However, to receive feedback, entrepreneurs depend on the input of others. To overcome this dependency on verbal input, entrepreneurs emphasize the role of learning by observing and comparing. Social comparison is the drive of individuals to evaluate themselves by observing others (Festinger, 1954). These social comparison processes emerge from metacognitive processes because it is the environment itself that works as an antecedent to think about thinking. For example, when entrepreneurs are pitching [C10]: *So I have seen other ideas that were not just great but were also very good at selling it at the competition, they were very good at communicating. So yeah we lost but we’ve learned a lot, we understood what we miss, and what we really have to work on a lot.* Another social comparison example can be seen in the case of [C2]: *After the start-up fair, we understood that we had to do much more work on the presentation, and how to behave in front of people you are presenting to.*

In both cases, the respondents understood that they had to improve their abilities because an emerging process took place where social comparison activated a process of re-thinking about their current abilities. This process of becoming aware as a result of the use of social capital is not evidently a straightforward process for everyone. Nascent entrepreneurs must adopt a willingness to implement these re-thinking processes, as [C8] states: *Another limitation that I have as founder, it is difficult, if you think about an elevator pitch, to convince people. I cannot do that, and I probably don’t want to do that.* The findings show a pattern in which engaging with others stimulates metacognitive processes, because the input of others, either by social comparison or feedback, causes a process of re-thinking about initial thoughts. These comparisons may be particularly present when nascent entrepreneurs make use of their social capital because it stimulates metacognitive processes in which individuals become aware of the skills in which they are inexperienced, as is shown in the start-up creation process of [C5]: *We are actually scientists, we are free from thinking of the economic reward. I am totally honest so I follow someone with more experience, with more initiative.* Importantly, this process of social comparison develops further in the steps start-ups take after the SUC. Here, a process takes place in which intentions to consider social ties utilizing observation convert into actionable decisions, such as concretely building a network around the start-up. For example, in the case of [C5]: *So it is great to take the awareness, from a point where the product is not ready yet, closer to the market, with the help of others. And we took the decision, I have to admit, thanks to the company we are currently working with. In fact, I have big news for you, I don’t know if you know the innovation site, where there is a research institute. So it is an innovation hub, with other companies. So here, they suggested to us a collaboration, and they agreed on the first testing sessions. If everything goes well, and it is promising enough for them, they would like to take about 15 orders from us.*

Additionally, adoption of feedback and social comparison processes emerge as a motivational trigger for entrepreneurs to engage further in local entrepreneurial ecosystems. The results show that, once respondents are aware of the added value of receiving feedback and learning from others, the willingness to search for additional networks, structures, and memberships significantly increases. An example of this emerging process can be exemplified with the case of [C1]: *Another person in our incubator, who is 50 years old, suggested us an exercise where we tell each other one good thing and one bad thing to each other. When you do this in a quiet situation, it feels better than when you discover it later in a complex environment. It really helped us to become a better team, to become aware of the things by others we are not so good at […]. The incubator in that sense is a very nice way to stay connected to the entrepreneurial environment. I feel that everyone can learn something from someone else, regardless age or expertise.*

During the second interview, [C1] evidently shows how valuable input received from feedback and social comparison contributes to the development of a local entrepreneurial ecosystem: *I think that the main decision we took was when we had the awareness about the ‘second solution’, our technical solution, because this was a result of an engineering project during the lockdown period, a society helped us. It was very good from a technical point of view, but it wasn’t still ready to market from an economical point of view. So I think that we were right, we took the right decision not to focus only, not to just stay on our engineering point of view, but to try to move forwards and collaborate.*

What emerges here is a process loop where entrepreneurs who recognize the usefulness of observing and listening to others, progressively enlarge their local entrepreneurial eco-system. This process is stimulated because the input of others operate as a metacognitive process for entrepreneurs, it encourages thinking about one’s thinking.

### Outsiders’ view consideration

A third aggregate dimension refers to the capacity of nascent entrepreneurs to consider an outsiders’ perspective. An outsiders’ perspective is the process in which entrepreneurs learn to think like an outsider when thinking about their own business, a crucial condition for innovation, growth, and success (Ensign & Robinson, 2016). This is because the consideration of an outsiders’ perspective generates more alternatives during the decision-making processes. [C1] for example states: *There is the risk to close your eyes if you only focus on what’s good for you, you have to ask yourself what’s good for your client. There is a risk when you focus only on the technical part of the start-up.* Metacognitive processes emerge here into beneficial outcomes for entrepreneurs because thinking about thinking processes promote the exploration of alternative explanations and open the possibility for various possibilities in problem-solving and decision-making (Graber et al., 2012). For example, during the process after the launch of the business, [C1] declares that he now sees his ability to consider the perspective of his potential clients as a passion: *I now found [after the SUC] networking with other people as a passion for me, the power to be an and think like an open-minded person, I like to make the links. To keep a possible customer focusing on our solution. For example, if you send an email to someone, and you never send a reminder, they will never tell you anything. So if you give a reason to call them, or to talk with them about something else, and then you get back to our product, you can facilitate the marketing part.*

The findings show that metacognitive processes have additional benefits for nascent entrepreneurs because they stimulate the growth of an entrepreneurial mindset. For example, [C8] states that: *When you pitch that idea to someone from a different environment, with a different mindset, you actually get a real clue of how valuable that idea is.* While [C10] confirms that using their network to add another co-founder with business expertise led to new ways of thinking about entrepreneurship after the launch of their start-up: *After we took [N] on board, we grew a lot as business persons. Before we just met to discuss the technical stuff, now we are looking more business-wise, we were not thinking about how a company worked before.* Here we see that, previously, [C10] did not think about his thinking to take the next step toward a business approach. It was the use of social capital that added the consideration of an outsiders’ perspective to the firm. Metacognitive processes take place here because it stimulates nascent entrepreneurs to become aware of what expertise is missing so that this can be sought elsewhere.

However, this process is particularly challenging for nascent entrepreneurs. For example, in the case of dropout [C6], we observe difficulties in the process of considering the view of outsiders: *I am generally unconscious of what I am doing, I was [during the start-up fair] really focussed on the project, on the main idea, and so I was pretty sure that my idea was better than other ideas. You know, my world is different, in the university world, you have time to talk about things. So the main problem was that people when we exchanged the idea, a lot of time they didn’t get it, they didn’t get what was the deal of my idea.* Here, indications from outsiders are not cognitively processed into re-thinking processes, resulting in an entrepreneur being unable to engage with others. What can be observed here is a lack of metacognitive processes, because the entrepreneur does not consider the possibility to re-think or re-consider his own conscious mind. Rather, the problem is profiled on others. As a result, these lack of metacognitive processes were responsible for an absence of considerations of alternative reasons why potential customers and partners didn’t engage with the entrepreneur.

### Dynamic interactions within the team

The different aggregate dimensions are deeply intertwined, as the previous quotes seem to confirm: an outsiders’ perspective affects the decision-making process of the entrepreneurial team as well. This happens because metacognitive processes stimulate nascent entrepreneurs to recognize the value of a diverse team, and this improves the balance in their collective decision-making processes.

The social ties that were built up during the development of the start-up were further engaged in the exploitation steps after the SUC, [C11] declares: *I now realize that the social aspects were really nice. I started to say ‘our’ project, you know, the small things. ‘We’ have to decide that, all these things, and they immediately felt part of the project, immediately. So one of them is working on one of my projects now. Right now she cannot do something [due to COVID-19], but it is my plan that at the moment that we can produce, to involve her, because I think that she is really good, she wants to learn a lot, we have a really good relationship.*

When individuals think about their thinking, they identify their unknowns, and this process consequently leads to the realization which abilities one has and of which are still missing. The findings show that different levels of self-confidence in a team are helpful to serve as a process of rationalization because the differences in thinking patterns within the team allow a process of identifying knowns and unknowns. This is an important finding since high levels of confidence are associated with processes in “which people do not know what they do not know” (Forbes, 2005, p. 624). For example, in the case of [C3]: *You know, having all the same idea…hmm, not a good idea. Usually, it is good to have some disagreements, to have better products, and to produce better thinking.* And [C1]: *In our case, there are the more optimistic and the more pessimistic ones. I am more in the middle, the main reason why I am like this is that I don’t want to be too opportunistic about the outcomes, for our project, before we really build it, it is my character.*

These findings emerge into outcomes that impact collective decision-making because metacognitive processes within the team are causing discussions in which different opinions are considered. For example, when [C5] states: *Clearly it is useful to communicate all together, if you are only focused on the solution without an idea, without considering other comments that can arise from discussions, you have no idea and will never find the real trade-off. So the trade-off gives every member a nice panoramic view of the project.* Different characters and experts within the team cause thinking about thinking processes that advance the decision-making process of the team. This occurs by differences in opinions, disagreement, optimism, or, as in the following example, expertise. Moreover, it implies that a diverse entrepreneurial team stimulates metacognitive processes, and the awareness of these differences are increasingly valued over time, such as in the case of [C10]: *For example, when [N] says something about strategy, of course for me that is like ‘okay, she saw this maybe 100 times and if her experience taught her so, I will take it as a valid feedback, validated by experience to interpret the information.’ So the difficulty is to understand and to give up some ideas you have sometimes. You might think it’s the right way, but when you interact with others and you get their feedback, you will understand that there are other ways. Maybe better, maybe worse, but there are other ways*.

However, a heterogeneous team needs to find an equilibrium in order to benefit from metacognitive processes. For example, in the case of dropout case [C4], who recently had changes in his team, states: *Basically I was looking for people that like brainstorming, with a creative mindset. […] The thing is, I don’t think that they are all so business-oriented. It’s nice to work with them but I don’t think that they are that motivated. People are not really work-oriented, this is what I observe and experience.* In the case of [C4], no trade-off has been found between different characteristics in the team to create a heterogeneous team. Too many same-minded individuals were part of the team, creativity was dominant, which made the stimulation of metacognitive processes problematic. On the other hand, in the case of [C10] above, the original team of founders was composed by similar abilities without an entrepreneurial background. The decision to recruit a member with entrepreneurial experience improved the collective team decision-making progress. It initiated a process in which the other members of the team started to re-think and question their original assumptions. As a consequence, these metacognitive processes created alternative ways of how the individual team members understood solutions in their decision-making process.

## Discussion

**Fig. 2:**
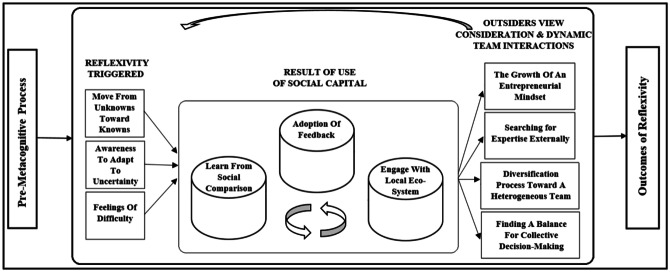
Empirical model

In order to advance theoretical insights, we follow Gioia et al. (2013) by developing, from our data structure in Fig. 1, an empirical model to be discussed in this section (Fig. 2). The model considers that nascent entrepreneurs start from a condition of pre-metacognition, in which they are not aware of what they do not know. Under these circumstances, their decision-making can be severely flawed by unknown unknowns. The topic has been overlooked by academic research, and this research aims at addressing a call for studies in this area, since “unknown unknowns in strategy may further our understanding of the consequences of heterogeneity” (Ehrig & Foss, 2021, p. 4).

The results show that reflective practices facilitate a process of change. Indeed, reflection as a driver of change is successfully promoted by metacognitive processes because it stimulates the awareness of one’s beliefs (Muis, 2007). The results indicate that these reflective processes serve as antecedents to creating awareness among nascent entrepreneurs for the purpose of understanding what they do not know (Petersen et al., 2008). These processes are particularly important for nascent entrepreneurs because they often lack the expertise to identify what they do not know. Later on in the process, entrepreneurs “start to understand what they do not know” and “what they need to know” to overcome their lack of expertise (Niittymies & Pajunen, 2019, p. 6).

On the one hand, reflection is triggered by an individual process, which leads to the creation of personal awareness in order to prepare nascent entrepreneurs for uncertain, unknown difficulties. The experience of feeling difficulty during the start-up process is important for the self-regulation function of metacognition. This is because feelings of difficulty have been found to alert individuals to make an effort in their decision-making process (Efklides, 2008). These cognitive cues are particularly important in the findings of this study because thinking about one’s difficulty provides entrepreneurs the awareness to prepare for unknowable, uncertain moments.

On the other hand, particular elements of social capital are crucial as input to revitalize metacognitive processes, because the input of others stimulates entrepreneurs to think about their thinking, such as when respondents received feedback. This complements previous findings on social capital since it has a positive effect on start-up progress for nascent entrepreneurs (Davidsson & Honig, 2003).

The outcomes thus highlight a reflective process with a central role for the use of social capital, an important condition to form start-up growth aspirations (Liao & Welsch, 2003). This research adds to the rich literature existing about social capital in entrepreneurship (Anderson et al., 2007) because it provides an alternative perspective, as represented by its role in metacognitive processes, in which entrepreneurs think about their thinking. These processes lead to new thinking processes that support nascent entrepreneurs’ awareness about difficulties they have not thought about before (Efklides, 2008). Additionally, these cognitive processes emerge into fruitful entrepreneurial outcomes, such as the growth of an entrepreneurial mindset and the necessity for nascent entrepreneurs to search for expertise externally to complement their often inexperienced entrepreneurial team.

An entrepreneurial mindset has been described as an “ability to rapidly sense, act, and mobilize, even under highly uncertain conditions” (Ireland et al., 2003, p. 967) and conceptualized into a situated metacognitive model in which entrepreneurial thinking is explained by individual differences in motivational and environmental interpretations (Haynie et al., 2010). This study extends these conceptualizations by empirically modeling a dynamic process in which social capital reinforces the entrepreneurial mindset, and specifies environmental conditions that lead to specific entrepreneurial outcomes when nascent entrepreneurs are in the process of launching their start-ups. This transitional process eventually leads to a diversification of the entrepreneurial team and a balance in the collective decision-making processes, highlighting the important contribution that metacognition is not purely an individual thinking process.

This study shows specific elements of social capital that are in a constant loop, in which nascent entrepreneurs reflect and learn from others with the help of comparisons and feedback. Consequently, the results show that this leads to further engagement with local entrepreneurial ecosystems, such as an incubation process in which nascent entrepreneurs actively engage with other entrepreneurs, mentors, accelerators, and business consultants. These relationships are crucial to establishing because they have a positive influence on firm survival (Mian, 1994).

The role of specific components of social capital in the metacognitive processes of entrepreneurs is another significant contribution of this study. Recent psychological perspectives on metacognition have emphasized that metacognitive processes are continuously updated “through observation of one’s and other’s behavior/actions and their outcomes” and “through communication and interaction with others” (Efklides, 2008, p. 279). The results of this study point to the specific role of feedback and social comparison because these serve to reinforce additional metacognitive processes and further engagement with others. The central role of social capital in the empirical model is justified by the lack of expertise of nascent entrepreneurs in the entrepreneurial process, which can be compensated and complemented by expanding involvement with external stakeholders, incubators, and other experts. Metacognitive processes stimulate extending expertise because thinking about thinking processes gives rise to the consideration of alternative options that create different types of opportunities (Gustafsson, 2004). The results of this study highlight the utilization of expertise as a result of metacognitive processes, extending previous findings through a model that highlights extending expertise as an additional advantage for thinking about thinking processes that is stimulated by engaging with others.

Haynie et al. (2012) have previously shown that individuals with higher degrees of metacognition use cognitive feedback more effectively. This paper finds that feedback is likely to come from interaction to the extent to which entrepreneurs leverage on human relationships inside and outside their venture (Markman et al., 2007), or from the extent to which individuals benefit from comparison within their social structures (Baron, 2004). Thus, we extend these previous findings in which this study shows empirical evidence of how nascent entrepreneurs, by using their metacognition, extend their social capital and go beyond their social networks, structures, and memberships to search for expertise externally. Hence, these emerging concepts lead to the growth of an entrepreneurial mindset, a crucial condition for nascent entrepreneurs to develop because the business success or failure highly depends on business skills such as communication, negotiation, perseverance, and the coordination of social ties (Lamine et al., 2014).

Individual differences between the respondents of this study showed that while some nascent entrepreneurs relied on previous business experience, others relied solely on their academic experience when launching a university spin-off. These differences are important to consider. For example, academic entrepreneurs have been found to lack the willingness to grow and do not seek profit maximization (Hesse & Sternberg, 2017). Additionally, the process of mobilizing resources can take years for academic entrepreneurs because these requirements are usually extensive (Druilhe & Garnsey, 2004; Garnsey, 1998). These discrepancies highlight the need for academically orientated entrepreneurs to develop a mindset that supports the mobilization of resources to further business growth. Metacognition has been found to stimulate this process because it is a cognitive resource that leads to the development of an outsiders’ perspective which helps nascent entrepreneurs re-think current strategies (Ehrig & Foss, 2021), and understand which expertise may benefit their start-up activities (Haynie et al., 2010).

Furthermore, this study proposes that metacognitive processes help in a better understanding of specificities in the dropout cases of this study. This assumption is grounded on the important conceptualization of Haynie et al. (2010) in which the authors reason that metacognition represents a heterogeneous learning process that explains why some individuals adapt to their context, while others do not. Since the empirical study has been conducted in two rounds (during the startup competition and after some time), the researchers had the opportunity to identify dropouts from the original entrepreneurial project. The results of this study show that the dropout cases showed little adaptability to feedback, and little effort to engage with their social capital during the process. Those who abandoned business after the SUC were occasionally composed by a rather homogenous team that generally perform better on routine tasks (Schjoedt & Kraus, 2009). Our findings highlight the importance for individuals with different backgrounds who are inexperienced in the entrepreneurial process to develop and stimulate thinking about thinking processes to learn to think like an outsider, an important condition for business growth and success (Ensign & Robinson, 2016). The empirical model of this study emphasizes an important transition toward a diversification of the entrepreneurial team and a better balance in the collective decision-making processes.

Unlike frameworks that have described entrepreneurship as an individual activity (Shane & Venkataraman, 2000), recent studies place entrepreneurial teams at the core of entrepreneurship, and as the responsible driver for most start-up activities (Harper, 2008). The results of this study show that metacognitive processes stimulate nascent entrepreneurs to search for expertise externally. This awareness can lead, as a consequence, to a diversification of the entrepreneurial team, as well as to a search for external advisors. Since heterogeneous teams perform better when operating in a novel context (Schjoedt & Kraus, 2009), the transformation toward a heterogeneous composition is particularly important in the decision-making process of nascent entrepreneurs. Indeed, effective decisions are more likely to come from heterogeneous teams because they consider more options when making decisions (Eisenhardt & Schoonhoven, 1990). This study proposes that the team composition has a significant effect on metacognitive processes because teams that are composed by differentiated members increasingly stimulate thinking about thinking processes.

Finally, the model shows that, when entrepreneurs think about their thinking, they become aware of their unknowns. Consequently, this process leads to the identification of missing resources that would complement the entrepreneurial team. It also leads to a more balanced collective decision-making process (West, 2007). For example, when entrepreneurs make decisions, they have been found to display higher levels of cognitive bias than managers (Busenitz & Barney, 1997). Cognitive biases arise because individuals rely mostly on their intuition when making decisions. A more rational, slow processing of information decreases biases in decision-making, but requires more cognitive effort, and is impracticable to be used most of the time (Kahneman, 2011). When decisions are collectively made, metacognitive processes improve decision-making processes. That is because metacognition engages with analytic reasoning and decouples itself from intuitive judgments (Croskerry et al., 2013). For example, self-awareness associated with improved metacognition diminishes cognitive biases in decision-making (Sadler-Smith & Shefy, 2007). The findings of this study suggest that, when nascent entrepreneurs make decisions collectively, they avoid relying solely on their own, limited expertise. This is because metacognitive processes provide decision-making advantages such as seeking out alternative explanations and exploring the consequences of these alternatives (Graber et al., 2012). Consequently, this outsiders’ view consideration has a crucial impact on the process of improved decision-making, because collective reasoning between individuals leads to interactions that facilitate disagreements and different perspectives on the decision-making process (Schraw & Moshman, 1995).

As Fig. 2 shows, metacognitive processes lead to outcomes, in terms of effective start-up from the original business idea and subsequent survival and growth. Our empirical study has a longitudinal nature, covering the period from the SUC to the effective startup of the venture, and thus permits to understand the role of metacognition in dropouts versus effective launches. This role seems particularly relevant, thus contributing to the entrepreneurship literature in this field, by adding the scarcely explored metacognition perspective.

## Theoretical implications

This study aims to contribute to entrepreneurship literature by uncovering the understudied role of metacognition in discussing how nascent entrepreneurs can deploy metacognitive processes in their start-up process. The research has found an important role of social capital when entrepreneurs think about their thinking. The comparison between two specific research contexts (the start-up competition and the following launch of the venture) offers a unique insight into how differences in the use of social capital lead to different metacognitive responses, that consequently impact nascent entrepreneurs and their teams in the decision-making process. These findings extend previous studies on metacognition by providing specific environmental elements that give rise to specific entrepreneurial outcomes (Haynie et al., 2010). Additionally, we offer new insights between metacognitive processes and decision-making, highlighting the role of metacognition in activating slower, deliberate, effortful thinking (Croskerry et al., 2013).

Moreover, this study is one of the first to use a qualitative approach to explore metacognition. Thus, we offer new opportunities for new explorative works to further investigate the antecedents and consequences of metacognition in the entrepreneurial process. Finally, the results expand our understanding of metacognition encompassing the role of collective cognition (West, 2007). This is because metacognition is not only related to the self, but also to others (Efklides, 2008), thus suggesting metacognition to be part of a collectively constructed process that has important advantages for the decision-making process.

## Managerial implications

The findings also contribute to pedagogic and educational practices. For example, metacognition can be trained and learned in classroom settings (Nelson, 1996; Nietfeld & Schraw, 2002). Thus, metacognition may be used as a practical tool that can be taught and learned, with the potential to have educational value to entrepreneurs and managers. Entrepreneurship education might benefit from this, given the fact that in-class training may lead to more self-reflective and self-regulatory results in an entrepreneurial context. Thus, metacognitive training may be implemented in entrepreneurship courses, accelerator programs, and incubators. These exercises may consequently lead to the execution of randomized experiments. For example, metacognitive training instructions may be applied as an intervention in decision-making practices. Since metacognition has been suggested to lead to cognitive improvement (Croskerry, 2002) and analytic forms of reasoning (Alter et al., 2007), experimental methods may test the use of a metacognitive checklist or exercise where managers or entrepreneurs may engage in reflexive and awareness exercises. Fruitful new insights may derive from the idea that training by definition is an applicable treat in such a session. Consequently, workshops could use these insights to design tools in which managers would be trained to use thinking about thinking strategies a priori a decision is made, in order to not fall prey to well-known managerial decision-making biases such as confirmation bias and anchoring (Bazerman, 1994).

## Limitations and future research suggestions

This paper is not without limitations. Firstly, the study uses a limited number of cases, thus preventing fully generalizable conclusions. To overcome this limitation, future studies might include different samples in their analysis, such as studying start-ups that are competing in different countries within different competitions over time in order to broaden the results of this study. This work allows for additional future research. For example, previous works have suggested that applying cognitive adaptable decision-making tools, such as metacognition, allow individuals to be both self-reflective and self-regulatory (Haynie et al., 2012; Hitt, 2000). This function of metacognition may help decision-makers to mitigate certain cognitive biases. Future research therefore might focus on the mitigating role of metacognition on cognitive biases. Knowing that entrepreneurs might be unreasonably confident, metacognition may help to avoid costly errors and could have benefits on new-venture performance (Mitchell et al., 2014). Also, work on metacognition has mostly focused on cognitive aspects, while just very recently other facets of metacognition have been highlighted, such as affect (Ustav & Venesaar, 2018). Future research could explore the role of meta-affection in entrepreneurship and explore its role with metacognition. Finally, although this work acknowledges that metacognition is beneficial for nascent entrepreneurs, simultaneously, too much thinking may lead to doubt, delayed decisions, or no decisions (Roy & Zeckhauser, 2015). Future research might thus investigate under which circumstances thinking about thinking may support entrepreneurs in their decision-making process, and when it may not. For example, perhaps that too much thinking about thinking may lead an entrepreneur to stay stuck in the intention-action gap. These future directions have great potential for entrepreneurship researchers.

## Conclusion

There is a recognized gap in studies in understanding how unknown unknowns affect firm capacity to adapt to change (Ehrig & Foss, 2021). This research focuses on nascent entrepreneurs, who may be even more affected by unknown unknowns in their start-up process, and how self-regulative mechanisms, such as metacognition, can support them. We thus contribute to understanding better which processes contribute to “enhancing the quality of new firms”, which has been recognized as a key issue for practitioners and policymakers, who share the objective to reducing the high failure rates of new ventures (Brixy et al., 2013, p. 157).

In this study, a sample of new venture initiatives was analyzed over time, in two distinct phases of their life: a startup competition and the subsequent launch of their venture. The findings contribute to new empirical insights about entrepreneurial metacognition, uncovering a reflective process with a crucial role for the utilization of social capital. Consequently, these metacognitive processes generate important outcomes for nascent entrepreneurs to move beyond the status quo, such as expanding local entrepreneurial ecosystems and growing an entrepreneurial mindset. As a result, these processes emerge toward dynamic team interactions that stimulate diversification, and improve collective decision-making processes. We also find that, when metacognitive processes are not enough developed, dropout from the original entrepreneurial project occurs.

## Data Availability

all materials and collected data will, upon request, be provided.

## Appendix

**Table 2:** Information about interviewees

*Case*	*Type* ^*a*^	*Sector*	*Market*	*Description*	*SUC result*	*Co-founders*	*Type of experience founder(s)*	*Dropout*
*C1*	Start-up	Sustainable Energy	B2B	A device capable of obtaining energy from vehicular traffic	3^rd^ place	3	Academic & Business Experience	No
*C2*	Academic spin-off	Food & Beverage	B2B & B2C	Sensors for naked-eye monitoring of meat spoilage	2^nd^ place	4	Academic Experience	No
*C3*	Academic spin-off	Life Science	B2C	A system for large-scale kinetic analysis	5^th^ place	0	Academic Experience	No
*C4*	Start-up	Consumer Goods	B2C	A compact dishwasher that washes dishes with 4 L of water in 1.2 min	No award	1	Business Experience	Yes
*C5*	Academic spin-off	Automation & ICT	B2B	Optimized machine learning algorithms with quantum speedups	No award	1	Academic Experience	No
*C6*	Academic spin-off	Biotech	B2B	A cryptographic technique based on protein folding	4^th^ place	1	Academic Experience	Yes
*C7*	Start-up	Digital Media	B2C	A platform and an app whose purpose to find caregivers and services	No award	0	Academic Experience	Yes
*C8*	Start-up	Eco-Solutions	B2B	A recycling process for sustainable development	No award	0	Business Experience	Yes
*C9*	Start-up	Food & Beverage	B2B	Sustainable sake in Europe	1^st^ place	2	Academic & Business Experience	No
*C10*	Start-up	Food & Beverage	B2B	An IoT platform for digitalized monitoring of beer drafting	No award	2	Academic & Business Experience	No
*C11*	Start-up	Web & Digital	B2C	An e-commerce site requesting and offering unusual products	No award	0	Academic & Business Experience	No

^a^Academic Spin-off has been labeled when the individual created the idea as part of his/her research at an university

**Table 3:** Interview scheme

*Topics*	*Purpose & Focus*	*Questions*	*Literature*
*Entrepreneurial Process*	Understand background entrepreneur and process: *When, where and why did the entrepreneur come up with the idea, what is the motivation, how was the decision-making process, previous experience in launching a start-up*	How did you come up with the idea, when did you start it, and why? What were your reasons and motivations to start this business? Do you have experience with the start-up world as a person? Can you give me an introduction of your start-up, how did you start? Where are you now, what are the differences between the start and now? Which decision did you make during the process? What are your objectives during this period?	McMullen and Dimov (2013); Hindle (2004)
*Teams & Team formation*	Differences in the founder team: *how and why was the team formed, did they start together, did they face difficulties, conflicts and how was it solved, how do they evaluate each other, which decisions and judgments were made?*	Can you tell me something about your team? How do you evaluate each other, do you use certain tools? Were there any conflicts in your team so far, and how did you solve them?	West (2007); Mol et al. (2015)
*Metacognition*	Understand how founders control and reflect upon their learning: *what they recognize about themselves, tasks, situations and their environment, understand the perception of own knowledge and competency and which ways or tools (de-biasing) they have to evaluate themselves?* COVID-19 context: compare SUC cases with the current context, and learn from start-ups that coped with uncertainty during the pandemic and how they approach this	Can you name me a specific task you are responsible for? Can you reflect on your role? How did your environment react on your input so far? Have you considered their perception? How do you reflect on these processes? How do you evaluate yourself? What was your initial forecast/expectation to achieve this year? Looking back at the competition, would you have done something different? How do you reflect on this process? What decisions have you made or/and not made because of COVID-19?	Flavell (1979); Haynie et al. (2010); Mitchell et al. (2014); Shepherd (2018)
*Social Comparison & Team Cognition*	The pursuit of new value through entrepreneurship is motivated by social comparison: One way to learn is by observing the behavior of other people (de-biasing)	Is it important for you to talk with your shareholders? How do you observe your competitors? How did you observe the students at the fair of ideas to recruit them? What are you hoping to get from your team? What do you know about the other student teams?	Mitchell et al. (2007); Festinger (1954)

## Footnotes

1 We refer the reader to the appendix for details about the cases.

## Code availability

N/A.
